# Editorial

**Published:** 1849-11

**Authors:** 


### THE MEDICAL EXAMINER.

PHILADELPHIA, NOVEMBER, 1849.

### CRYPTOGAMOUS ORIGIN OF CHOLERA.

In the London Medical Times of September 29th, 1849, the following curious microscopical discoveries are published under the editorial head.

"It appears, that Dr. Brittan has discovered in the flocculent sediment which exists in the matter vomited and voided per anum in cases of cholera, (and which has been variously considered to be mucus, fibrine, or another protein compound in a peculiar and as yet undetermined state,) certain organic corpuscles, which both to him and to other observers well versed in such inquiries, appear to be fungi. In their most developed condition these bodies are of some size, that is, are considerably larger than blood corpuscles. They have a well-defined transparent double outline, which occasionally becomes opaque and thickened. The centre is occupied by granular matter or by cells, and has somewhat of a yellowish or brownish hue. The smallest of these bodies are much more minute, and require the highest powers (such as Ross's or Powell's 12th) before they can be satisfactorily made out. They then appear as minute cells, with a clear double outline, and bear a very strong resemblance to the sporidia of fungi. Both kinds of cells appear to be spherical, or but slightly flattened, and the transition from the one into the other can be traced with a little care. These bodies are not to be confounded with the granular cells which Dr. Brittan also finds in great numbers.

Did the observation stop here, the discovery of fungi different from the common torulæ, which often form rapidly in cholera stools, and which have been figured and described by Boehm and others, would, no doubt, if confirmed, be highly important. But Dr. Brittan has proceeded further. By causing the air of rooms and districts in which cholera is prevailing, to pass through a glass tube surrounded by a freezing mixture, he has condensed the atmospheric watery vapor. In this vapor he finds under the microscope, minute spherical bodies, corresponding as far as the eye can determine, most completely with the smallest kinds of the presumed fungous cells, which can be noted in the cholera dejections. We understand that observers of the highest eminence in this department of Histology, have expressed a decided opinion as to the identity of these bodies.

We have first to determine what these bodies are; since, of course, it is by no means certain that they are fungi. But, supposing they *are* fungi, they may merely be products; low cellular forms developed rapidly in the favorable matrix of the cholera stool, just as the little vibriones are, which sometimes can be seen under the microscope moving in shoals in a dejection which has only the moment before been passed from the body. Perhaps the spores of these fungi may exist at all times in impure and contaminated air, and may merely find in the cholera discharges their appropriate blastema. We foresee the greatest difficulty in deciding upon some of these points, and, assuredly, the only accurate method will be, to make at once an inquiry as extended as possible into all the questions connected with the subject.

These microscopic observations have been confirmed by those of Dr. Alison, of Birmingham, Mr. Swayne, of Bristol, and Mr. Curme and Dr. Cowdell of Dorchester. The latter gentlemen have also observed in the clammy sweat accompanying the last stages of collapse in cholera, as well as in other excretions, minute organized bodies closely resembling those admitted by naturalists and microscopists to have a *protophytic* organization.

The priority of this discovery admits of some question, inasmuch as there is abundant evidence that cryptogamous growths had been seen in cholera dejections at a period anterior to the observation above recorded. In the "Treatise on Physiology and Pathology for Physicians and Naturalists," published at Leipsic in 1843, occur these words, "Böhm fand Pilze im Darminhalte der Cholerakranken." Boehm discovered cryptogamous plants in the dejections of cholera patients." Of these growths he published accurate drawings.

But, waiving for the present the discussion of priority, there are some other elements to be taken into consideration before the establishment of the fungous origin of this, or any other disease. It must be proved that these growths are invariably present in cholera dejections; that they exist in the atmosphere of infected localities; that they are uniform in their character and appearance; *and that they are met with under no other circumstances.* Should these points be established, they would be strikingly confirmatory of the theory long ago advanced by Prof. Mitchell of this city, and since him, by Dr. Cowdell of England, of the cryptogamous origin of this disease. At this early stage of the examination, however, any conclusion would be premature and ill-founded; it is only by the severe test of repeated observation and careful experiment that a theory such as this can be established. As was observed on another occasion, "the philosopher sets out with a preconceived idea, the result of profound thought and careful examination of every fact or phenomenon, that can have any—the most remote—bearing on the subject of his inquiry; he observes and compares the recorded observations of others, and if, after a sedulous examination into every possible source of fallacy, he finds that the observations confirm and establish his hypotheses, he correctly infers that he is justified in regarding that, which was at first hazarded as an hypothesis, to be a law of phenomena, and a solid addition to science. If, on the other hand, the result of reiterated observation of phenomena does not support—and *à fortiori*, if it negative his preconceived hypothesis—he unhesitatingly rejects it, and substitutes another, which has to be subjected to the same scrutiny; and thus he proceeds, until at length he succeeds in framing and establishing one, that receives unquestionable support from observation."

Some of the difficulties that present themselves in the present case arise from the fact that cryptogamous growths are found under other circumstances and in other diseases. Henle found cryptogamous plants in the vaginal discharges of a syphilitic patient; Schonlein, in the fluid of abdominal dropsy; Turpin, in the milk in the interior of the milk tubes; Quevenne, in the urine of a diabetic patient; Goodsir, in the matters ejected in pyrosis; and Klencke, in a sore in the interior of the nose of a woman in whose house toadstools grew.* Within a short period, also, Dr. Leidy, of this city, has discovered cryptogamic plants in the mucus taken from a stomach which had been softened by disease, and which were similar to those often found in the tartar of the teeth.

It is thus seen that fungi may be found in other diseases in man. But it is not to man alone that these parasites are confined, nor is the knowledge of their existence at all recent. In 1832 Mr. Owen read some notes before the Zoological Society on the anatomy of the Flamingo, (Phœnicopterus ruber,) in the lungs of which he found numerous tubercles and vomicæ, the inner surface of which latter was covered with a greenish mould or mucor. Mr. O. presumed these growths to have taken place during life, and thence concluded that internal parasites are not derived exclusively from animals, but that there are *Entophytes* as well as Entozoa. (Philosophical Mag., 1833, New Series, Vol. II., page 71.

In 1835, M. Bassi, of Lodi, and M. Balsamo, of Milan, discovered that the disease of the silk-worm, called *muscardine*, was owing to the growth on, or within the body, of a cryptogamous vegetation. (Annales des Sciences Naturelles, Vol VIII., new series, p. 229.)

In 1839 Schonlein announced the existence of *mycodermata* in the crusts of Tinea favosa, (Müller's Archives.) MM. Fuchs and Langen-

* Abhandlungen aus dem Gebiete der PHYSIOLOGIE und PATHOLOGIE für Aerzte und Naturforscher von Dr. P. F. Klencke, Leipzig.

beck, at Gottingen, supposed that they proved the existence of *mucores* not only in the crusts of Tinea favosa, but also in the majority of the eruptions termed scrofulous. (Traité des Maladies de la peau Gottingen, 1840.) Latterly much fuller accounts, and more correct descriptions of this disease have been given by Mr. Gruby of Vienna, who states that the crusts of Tinea favosa are made up of aggregated *mycodermata*. (Comptes rendus.)

In 1840 M. Des Longchamps described some layers of *mouldiness* occurring in the aerial cavities of the eider duck. (*Anas Mollissima.*) (Ann. des Sci. Nat., June, 1841, p. 371.)

In 1841 Mr. Westwood exhibited dried specimens of Chinese larvæ, from the back of the necks of which a slender fungus, twice as large as the body of the insect, had been produced. (Annals of Nat. Hist., Nov., 1841.)

MM. Rousseau and Serruriers discovered a mouldiness of a different character from that described by M. Des Longchamps, which is not unfrequently found in pigeons and fowls exposed to a damp, cold atmosphere. They found it also in a paroquet, which died of tubercular disease, in a sort of false membrane between the intestine and vertebral column, which membrane was covered with a greenish pulverulent mouldiness, so light and so little adherent, that it could be blown off as a fine powder. A similar affection has been noticed by him in animals of other classes, as in Cervus Axis and Testudo Indica. And Langenbeck noticed the development of highly organized members of this division of the vegetable kingdom, in the body of a patient who died of typhus. (Microscopic Journal, London, Dec., 1841.)

In some animals cryptogamous plants are invariably present in the intestinal canal. Dr. Leidy has shown us three distinct genera, not heretofore known, which he has discovered in the small intestine of the *Julus Marginatus* of Say, an animal somewhat resembling the Centipede, a description of which he presented to the Academy of Natural Sciences, of Philadelphia, at their last meeting. These plants, the largest which may be seen with the unassisted eye, he has called *Enterobrus Elegans*, or intestinal alga; *Cladophytum comatum*, or hair-branched plant; and *Arthromitus cristatus*, or tufted-pointed thread. They are not only found growing from the basement membrane of the mucous layer of the intestine, but even upon the bodies of parasitic entozoa that infest the same cavity.

The above briefly recited facts, too few in number to admit of any general deduction, yet make it clear—

1st. That parasitical growths occur in nearly all classes of the animal kingdom.

2d. That these growths usually arise on the surface of the animal organism, and are sometimes prolonged thence into the texture of the part.

3d. That they have, in several instances, been ascertained to constitute the cause of disease and death; and that the disease thus produced, has been found in some cases contagious.

4th. That they are probably of two kinds, the one peculiar to animal bodies, and the other consisting of those cryptogamic vegetations which readily sprout up under favorable circumstances, on almost any inanimate substance. To the former kind may be referred the muscardine of the silk worm and mycoderm of Tinea, and to the latter, most of the other growths above alluded to. (Op. cit.)

A few words only as regards priority of discovery. It has already been shown that microscopic fungi were discovered by Boehm in cholera dejections, during the visitation of that disease in 1832. In a letter dated June 6th, 1849, from Dr. H. Holland to Prof. Mitchell, Dr. H. states that Ehrenberg of Berlin, in his recent researches with the microscope into the various forms of organic existence in the atmosphere, published in the Transactions of the Royal Academy of Berlin, had tabulated nearly 400 species of such organic forms thus obtained. He was led to the research in relation to the phenomena of cholera, as the disease occurred in Berlin in the autumn of last year. "In the course of our correspondence," says Dr. Holland, "he sent me some remarkable specimens of what he terms the *Purpurmonade*, as it appeared about the 29th of October, in Berlin, very extensively tingeing the bread and other eatables, of a vivid vermillion color, corresponding doubtless with the *white* you (Dr. Mitchell) describe as coloring paste, starch, &c. in Philadelphia during the prevalence of cholera there in 1832. Though it is hardly possible, looking to material evidence, to associate this with cholera in any direct relation of cause and effect, yet is the coincidence remarkable in every respect."

Ehrenberg has found in this seemingly simple vermillion colored substance, two cryptogamic and one animal species. The latter he terms *monas prodigiosa*.

Without any previous knowledge of the observations of Ehrenberg, Dr. Burk, of London, obtained analogous results from a portion of the affected bread; viz., two vegetable forms and one animal; the latter he considers a species of vibrio. So much for the discovery.

The Editor of the London Medical Times, with a candor that does honor to him as an impartial judge, awards to Dr. Mitchell the credit of first advancing the cryptogamous theory.

"The priority of having first entertained the idea that cholera depended on the presence of fungi in the atmosphere must be accorded to Professor Mitchell, of Philadelphia, whose ingenious work on the cryptogamous Origin of Fever, Cholera, &c., we reviewed three weeks since. His views were embodied in a course of lectures delivered at the Jefferson Medical College during the session of 1846—1847. In the Introduction he thus addresses the gentlemen who, at that time, composed his class:—'To you I had the honor of delivering, nearly in their present shape, the lectures which I now send to the press. Previously, I had not put my ideas on the subject of which they treat into so formal a shape, although I had announced *for years* to each successive class, my impression that, possibly, the protophytes might afford a good explanation of the causation of malarious and other diseases of a febrile nature. Of the production thus, at least, of yellow fever and cholera, I entertained less doubt, and taught, therefore, the sentiment with less reserve.' And further on, he continues: 'Experiments are in progress, which seem to promise more direct and unquestionable proof of the validity of our hypothesis.'"

We have thus presented to our readers a brief and hasty examination of the subject in a few only of its aspects. Already does the "cryptogamous theory" gain ground. Dr. Wm. Budd has adopted it, and strongly urges a method not only of destroying the poison by receiving all cholera discharges into some chemical fluid known to be fatal to the fungous tribe, but also of preventing its dissemination by supplying water from an uninfected district, "As water is the principal channel through which this poison finds its way into the human body."*

The difficulties that we have presented are not intended to discourage investigations, far from it; but rather to invite that sedulous and unbiassed observation by which alone the doctrine must stand or fall. "The value of these observations necessarily depends upon the universality of the phenomena; but at present, perhaps not more than 100 examinations have been made of cholera discharges for the purpose of determining the presence of these 'annular bodies,' and these observations have been made in only one or two localities. But to prove these fungi, if they are fungi, to be the invariable elements of the cholera discharges, and of the cholera atmosphere, examinations must be made, not in hundreds, but in thousands of patients; not in two, but in hundreds of localities."† According to their own statements, Dr. Brittan and Mr. Swayne declare that in some even of the most severe and rapidly fatal cases, few if any of the "cholera cells" appeared in the discharges, while in others they were "few and small," "not many and small," and "very few and small."

* See the abstract of this letter in the London Lancet.
† London Medical Times.

We hope most sincerely that this subject will be examined, and that continued and more extensive observations will be made; but let us have *all* the facts before a principle is established, let us have a reason for the faith that is in us, and let us bear in mind the saying of Bischoff, "There never is an important and comprehensive discovery made at once; the elements of it are generally obtained from different quarters, and from all these truth at last results."

Note.—After the above article had gone to press, we had the pleasure of reading a letter from *Assistant Surgeon* Robert Southgate, U. S. A., to Prof. Mitchell, in which he describes the sudden appearance of intermittent fever in his own family, while stationed at Fort Gratiot, Michigan, coincidently with the development of a large quantity of fungi in the cellar of his quarters, which cellar had not been opened for a very long time. What is singular in this narrative is, that six ofother families numbering some thirty souls, (and residing in similar quarters,) and of two companies of Infantry at the same station, not one was attacked with fever of any description. We regret that we have not space to give the entire letter, which would amply repay the perusal. The above fact is all that our limits will allow. If it do not substantiate, at least it does not invalidate the theory of the "cryptogamous origin," and it is another item of testimony from a reliable source, in favor of a doctrine, which, if it do not lead to the desired goal, presents much to invite further researches in the same direction.

---

CARBONATE OF SODA AS AN ANTIDOTE TO THE CHOLERA POISON,

We invite the attention of our readers to the following documents, and will be pleased to learn the experience of any with the remedy proposed. The letter of Dr. Maxwell is an *exact* copy of that transmitted to the Department of State.

Dr. Francis G. Smith,

*Dear Doctor*,—I send to you in your editorial capacity the attending documents, just received from the Department of State in Washington. As they relate to an important subject, you can determine to what extent the idea of Surgeon Maxwell is well founded, and should be publicly noticed. There is, of course, no novelty in sub-carbonate of soda being good as a composer of the stomach in its irritable states. Its absolute ascendancy over Asiatic Cholera is, however, a different affair, and it, as prescribed by him, may be worthy of a special trial.

Very respectfully,

*October* 25*th*, 1849. 65* W. E. Horner, M. D.

DEPARTMENT OF STATE, *Washington, October 23d,* 1849.

Doctor Horner,

*Sir,*—I am directed by the Secretary of State, to forward to you the accompanying copy of a letter received with the despatches borne by the last steamer to this Department, from the U. S. Commissioner in China, Mr. Davis. Yours respectfully,

GEO. P. FISHER, Clerk.

---

*Hydra-bad, Deckan, August 25th,* 1849.

*Sir,*—I do myself the honor to communicate to you for the information of the President of the United States, and the benefit of the people, the important fact which I have just ascertained in the treatment of cholera, viz: that the carbonate of soda is a speedy and effectual antidote to the poison of that disease.

I give it immediately a case of cholera is brought, in doses of a teaspoonful dissolved in gruel or water, and drank as hot as the patient can drink it.

It allays the pain and burning of stomach, produces sleep, and restores the heat of skin and pulse in a very short time.

If it should be vomited, I immediately repeat it with a little laudanum, and a full dose of oil, *so as* to cause the antidote to pass down as speedily as possible to the poison in the small intestines.

When any portion of the oil and antidote is passed in the evacuations, convalescence *will be found* to have already commenced, the patient will presently *pass urine,* and *then be out of all danger.*

I continue the antidote morning and evening, (if necessary,) and reducing the dose.

I will not trouble you with details, which will appear hereafter.

By thus addressing the head of such an extensive empire, I make sure that the knowledge of this antidote will be speedily transported through its vast extent, instead of being left to chance to work its way up against the stream.

Besides I am only performing what I consider a duty, at a time when the epidemic appears to be on the increase.

And, with the greatest respect, I remain your most obedient and obliged servant, N. E. MAXWELL, M. D.

Surgeon, 3d Light Cavalry.

To the Secretary of State, United States, America.

---

DEPARTMENT OF STATE, *October 22d,* 1849.

The above is a true copy of the original on file in this Department.

JOHN M. CLAYTON, Secretary of State.

There is much evidence in favor of the saline treatment of cholera, originally proposed, we believe, by Dr. Stevens; certainly, at least, carried out with much success by him. Dr. Stevens states that of one thousand cases treated by him, or under his direction, not more than six per cent. have proved fatal. These cases included those occurring both in hospital and private practice. A letter from the Regent of Norway declares that the saline treatment was very successful. In Sweden it is stated to have been attended with better results than any other practice.

In the premonitory stages a seidlitz powder is given if there be diarrhœa; if sinking, without diarrhœa, be present, then a more active saline, as sulphate of magnesia, is used in combination with the Seidlitz powder, the patient drinking freely of beaf tea seasoned with common salt. In the second stage, or that of developed cholera, the following powder is administered:

"℞ sodæ sesquicarb., ℈j.; sodii chlorid. ʒj.; potassæ chloratis, gr. vii., misce—to be dissolved in half a tumbler of water, and administered in severe cases every half hour; in some malignant cases, every fifteen minutes; while in those cases which are not very severe, it is to be given every hour. The frequency with which the dose is to be repeated in each particular case varies according to its circumstances. In every instance, the saline must be continued until the circulation is fairly restored; when once that point is gained, the intervals between the doses may be lengthened; when re-action is completely established, it is to be left off by degrees. In extreme cases, the dose of the chloride of sodium is to be increased two drachms, and in some cases to even more than this. In those cases where the stomach is very irritable, a dilute solution of chloride of sodium is to be thrown up into the intestines; the temperature of this saline enema being as high as the patient can easily bear, which, as a general rule, is about 100 degrees Fahrenheit. When properly used, this is a means of great value. When the stomach is very irritable, the use of the saline powder may be occasionally suspended, and common effervescing mixtures, or small doses of the common soda powders, with an excess of the carbonates, used, until the vomiting abates, and then the carbonate of soda, with larger doses of the chlorate of potash, may be given without the chloride of sodium, or the chlorate of potash may be given by itself, in doses of ten grains each."

The plan of treatment recommended by Dr. Stevens, although not identical with, is very similar to that of Surgeon Maxwell.—Eds.

---

THE MEDICAL SCHOOLS OF PHILADELPHIA.

It is gratifying to be able to state that the classes now in attendance upon the lectures of the different schools are as large as we ever remember to have seen them at this period of the course. This, it will be remembered, it under circumstances different from former years.

The lectures in all the schools commenced some weeks in advance of the accustomed date; notwithstanding which fact, and other causes, such as sickness, death, &c., which would make it probable that many would be prevented from attending lectures, the number of students now in town appears to be fully equal to that of last year. We hope that this auspicious beginning may be crowned with success, and that the succeeding year may find full classes in attendance at the very commencement of the courses.

---

### APPOINTMENTS.

Dr. J. M. Wallace has resigned the lectureship on surgery in the Philadelphia Association for Medical Instruction, and Dr. J. H. B. McClellan has been appointed in his stead.

Dr. Wallace was one of the earliest members of the Association, and during his long connection with it his duties were performed with zeal and ability. He bears with him the best wishes and highest esteem of his colleagues, with their regrets at his departure from among them.

Dr. Henry H. Smith has been appointed one of the surgeons to *St. Joseph's Hospital*, Philadelphia.

---

### TRANSYLVANIA MEDICAL JOURNAL.

We have received the second number (but not the first) of a new Journal published at Lexington, Kentucky, bearing the above title, and under the auspices of the Transylvania Faculty of Medicine.

We heartily welcome the new aspirant into the editorial corps, and trust that his most sanguine expectations may be realized. We are certain of one thing, however, that he will find, as the late Dr. Butterfield said, that editing a medical journal, although pleasant work in the main, is very like having a note in bank—*it shortens the months amazingly*.

